# Sleep Quality in Medical Staffs During the Outbreak of Coronavirus Disease 2019 in China: A Web-Based Cross-Sectional Study

**DOI:** 10.3389/fpsyt.2021.630330

**Published:** 2021-06-09

**Authors:** Furong Jiang, Yi Xiao, Huixi Dong, Siyu Liu, Feng Guo, Zhicheng Gong, Shuiyuan Xiao, Minxue Shen, Qiuhong Zhou, Jianling Li

**Affiliations:** ^1^Department of Mental Health Center, Xiangya Hospital, Central South University, Changsha, China; ^2^Department of Social Medicine and Health Administration, Xiangya School of Public Health, Central South University, Changsha, China

**Keywords:** sleep quality, stress, axiety, depression, social support

## Abstract

**Background:** The aim of this study was to describe the sleep quality and its influencing factors among medical workers of different working statuses and staff types during the coronavirus disease 2019 (COVID-19) epidemic.

**Methods:** Through an online questionnaire survey, all medical staffs in Xiangya Hospital were invited to complete sections on general information, the Self-Rating Scale of Sleep (SRSS), the Depression, Anxiety and Stress Scale (DASS-21), the Social Support Rating Scale (SSRS), and the Simplified Coping Style Questionnaire (CSQ).

**Results:** A total of 4,245 respondents completed the survey. Among them, 38.7% had sleep disturbance. After matching, the SRSS scores in the staffs who were assigned to the intensive care unit (ICU) of Union Hospital in Wuhan and working in the epidemic area of Xiangya Hospital were not significantly different (*P* > 0.05); the SRSS scores in the battlefront staffs were significantly higher than (*P* < 0.05) those who were not treating patients infected with COVID-19. The SRSS scores of nurses were significantly higher than those of doctors and hospital administrators (*P* < 0.01). Anxiety, depression, and coping style were associated with sleep disturbance.

**Conclusion:** The sleep quality of the medical staffs has been impaired during the epidemic period, especially among nurses, doctors, and administrators who are working on the front line. Medical institutions should strengthen psychological services and coping strategies for medical staffs.

## Background

In the past decade, there have been several virus outbreaks: the 2009 H1N1 influenza pandemic around the globe, the Middle East respiratory syndrome-associated coronavirus (MERS-CoV) spreading throughout the Arabian Peninsula, the Ebola virus sweeping across West African, and the Zika virus endemic in the Americas (1). Recently, a pneumonia outbreak caused by a novel coronavirus that was detected in Wuhan, Hubei Province has spread across China and beyond since December 2019 (2). The WHO characterized coronavirus disease 2019 (COVID-19) as a pandemic on March 11, 2020 (3). As of March 22, 2020, a total of 332,930 laboratory-confirmed COVID-19 cases and 14,510 deaths were reported by 187 countries or regions around the world, with 40,788 confirmed new cases and 1,727 new deaths in the preceding 24 h (4). As the outbreak continues, health workers have been persistently confronted with heavy workloads and a high risk of infection. As of February 11, more than 3,000 medical workers were reported to be infected with COVID-19 (5). In this large-scale public health emergency, medical staffs are facing unprecedented physical and psychological pressures. Some studies have reported that mental and sleep problems have been prevalent among medical workers in Wuhan and other areas during the period. Zaki et al. (6) showed that negative emotions, such as anxiety and depression, were obvious in medical workers during the epidemic, and that 41% of hospital staffs had disrupted sleep/wake cycle after the quarantine. Wu et al. (7) showed that the main factors affecting the sleep quality of clinical infection control nurses included whether they had confidence to complete tasks, anxiety, and stress load. Chen et al. (8) found that insomnia symptoms were present in 34.3% of hospital workers, and that social support was a protective factor. It is relatively well-known that sleep affects both physical and mental health (9–11). As a result, good sleep quality is the premise of the physical and mental health of the medical staffs. During the epidemic period, there have been few domestic articles on sleep considering all sectors of the hospital staffs. The samples for this study were selected from representative hospitals in Hunan Province to study the sleep and related factors of medical staffs in various occupations. A series of prompt actions, such as the lockdown of Wuhan and even the entire Hubei Province, follow-up supervision, and timely reporting of information on the epidemic situation, early detection, strict quarantine, and provision of treatment with no personal charge for confirmed patients, was imposed by the Chinese government with the purpose of containing the outbreak. Furthermore, over 8,000 doctors and nurses from other provinces and cities, merging into 68 voluntary medical teams, converged in Hubei, the hardest-hit province in China, to combat the novel coronavirus in collaboration with the local medical workers (12). At the request of the National Health Commission, Xiangya Hospital dispatched 136 medical staff members to take over two intensive care units (ICUs) of the Union Hospital of Huazhong University of Science and Technology. Hunan is the neighboring province of Hubei, with a total of 1,018 confirmed cases being reported. As the largest top-class tertiary hospital in Hunan Province, Xiangya Hospital of Central South University has screened a significant number of suspicious cases at the local level by setting up two 24-h fever clinics and four screening wards, one for patients with respiratory disease, two for suspected patients with mild symptoms, and one for severely affected patients. In response to this disease, the medical staffs from the department of infectious disease, as well as the volunteers from other departments, such as the department of intensive care, respiratory medicine, and emergency medicine, were all summoned to join in the battle against the virus. To describe their sleep quality and identify related factors, we investigated all staffs of Xiangya Hospital through an online survey. The study aimed to provide evidence for designing and implementing interventional strategies for those who reported impaired quality of sleep.

## Methods

### Study Design and Participants

This was a single-centered cross-sectional study that investigated all medical staffs in Xiangya Hospital through an online (WeChat-based) survey. This study was approved by the Ethics Committee of Xiangya Hospital, Central South University. The questionnaire was distributed by the administrative department of the hospital from February 13, 2020 to February 15, 2020. Surveys were excluded if they were completed in <100 s or if 70% of the answers were identical.

### Sample Size Estimation

A *post-hoc* estimation of the sample size was performed. Assuming a significance level of 0.05, statistical power of 80%, and prevalence levels of sleep disturbance of 41.8 and 58.1%, respectively, for staffs assigned to Wuhan and those working locally (Table 1), the minimal sample size for chi-square test should be 49 for each group, which was satisfied by the current study.

**Table 1:** Demographics and characteristics of the study sample.

**Variable**	***n* (%)**
Gender	
Male	955 (22.5)
Female	3,290 (77.5)
Marital status	
Single	798 (18.8)
Married	3,339 (78.7)
Divorced	1,089 (2.5)
Age	
<31	1,368 (32.2)
31~40	1,818 (42.8)
41~50	714 (16.8)
>50	344 (8.1)
Educational level	
High school and below	52 (1.2)
Junior college	368 (8.7)
Undergraduate and above	3,825 (90.2)
Years of working	
≤ 5	1,080 (25.5)
6~10	1,528 (36.0)
11~20	807 (19.0)
21~30	588 (13.9)
≥31	241 (5.7)
Professional title	
Junior an below	1,797 (42.3)
Intermediate	1,946 (45.8)
Senior	502 (11.8)
Staff type	
Nurse	2,131 (50.2)
Doctor	868 (20.4)
Pharmacist	128 (3.0)
Technician	368 (8.7)
Administrator	395 (9.3)
Researcher	99 (2.3)
Logistician	155 (3.7)
Others	101 (2.4)
Working status	
Working in the non-epidemic area of the hospital	2,749 (64.8)
Working in the epidemic area of the hospital	263 (6.2)
Assigned to the ICU of Union Hospital in Wuhan	86 (2)
Working at home	347 (8.2)
Quarantined	92 (2.2)
Resting at home	708 (16.7)
Average monthly income(CNY)	
≤ 5,000	278 (6.5)
5,001~10,000	1,696 (40)
≥10,001	2,271 (53.5)
Disease history	
None	28,689 (67.6)
Had disease history	1,377 (32.4)
Living situation	
Living alone	613 (14.4)
Not alone	3,632 (85.6)
Exposure to confirmed or suspected COVID-19 cases	
Exposure	2,105 (49.6)
Non-exposure	2,140 (50.4)
Personal economic impact by the outbreak	
No impact	930 (21.9)
Have impact	3,315 (78.1)
Recognition of the effectiveness of protection	
Effective	2,655 (62.5)
Inffective	1,590 (37.5)
Concerns about insufficient supplies of protective materials	
No concerns	230 (5.4)
Mild concerns	882 (20.8)
Moderate concerns	1,912 (45.0)
Heavy concerns	1,221 (28.8)

### Measurements

The questionnaire included the following sections: general information, sleep quality, symptoms of stress, anxiety, and depression, social support, and coping strategies.

#### General Information

Sociodemographic characteristics included gender, age, marital status, educational level, years of working, staff type (doctor, nurse, technician, pharmacist, administrator, researcher, logistician, other), professional title, working status (working in the non-epidemic area of the hospital, working in the epidemic area of the hospital, assigned to the ICU of Union Hospital in Wuhan, working at home, resting at home, quarantined), disease history, living situation (living alone or not), exposure to confirmed or suspected COVID-19 cases, average monthly income, personal economic impact from the outbreak, recognition of the effectiveness of protection, and concerns about insufficient supplies of protective materials.

#### Sleep Quality

Symptoms of insomnia were measured by the Chinese version of the Self-Rating Scale of Sleep (SRSS), a self-reported scale that was tailored for the Chinese population by psychologists. It has been widely used in studies of the Chinese population, with validated psychometric properties and an established normative range, and the Cronbach's alpha was 0.6418 (13). The SRSS includes 10 items, and each item has 5-point Likert responses. The participant selects a number from 1 to 5 depending on how much the statement applied to him/her over the past month. Total scores can therefore range from 10 to 50. A global SRSS score >22 was classified as sleep disturbance. In this study, the Cronbach's alpha coefficient of the SRSS was 0.893.

#### Symptoms of Stress, Anxiety, and Depression

Symptoms of stress, anxiety, and depression were measured by the Chinese version of the short-form Depression, Anxiety and Stress Scale (DASS-21), which was first proposed by Lovibond and Lovibond (14) and later reduced to 21 items by Antony et al. (15). Studies by domestic scholars have found that the DASS-21 has high reliability and validity. When applied to college students, the Cronbach's alpha coefficients for the three subscales of the DASS-21 were 0.77, 0.79, and 0.76, respectively (16). There are seven items in each of the three subscales, with scores ranging from 0 to 42. A DASS-21 stress subscale score >14 was classified as stress; anxiety subscale score >7 was classified as anxiety; and depression subscale score >9 was classified as depression. In this study, the Cronbach's alpha coefficients for the three subscales of the DASS-21 were 0.887, 0.870, and 0.880, respectively.

#### Social Support and Coping Strategy

Social support was determined by the Social Support Rating Scale (SSRS), which was developed for the Chinese population by Xiao (17). The SSRS was utilized to assess the level of social support over the past year, including subjective support (four items), objective support (three items), and support available (three items). The total score ranges from 12 to 66 and can be classified into low-level support (≤22), medium-level support (23–44), and high-level support (>44). The SSRS has been validated to have favorable reliability and validity, Liu et al. (18) showed that the Cronbach's alpha coefficient of the SSRS was 0.896 and was 0.849, 0.825, and 0.833, respectively, for the three dimensions of the SSRS. In this study, the Cronbach's alpha coefficient of the SSRS was 0.836 and was 0.784, 0.770, and 0.747, respectively, for the three dimensions of the SSRS. Coping strategy was measured by the Simplified Coping Style Questionnaire (CSQ). This scale was compiled by Xie (19) and includes two dimensions: active coping (12 items) and passive coping (8 items). The items were measured using 4-point Likert scales (0 = ever; 3 = very often). The instrument has been commonly used in China and shows good reliability and validity (19). The Cronbach's alpha coefficient of the CSQ was 0.90 and was 0.89 and 0.78, respectively, for the two dimensions of the CSQ. In this study, the Cronbach's alpha coefficient of the CSQ was 0.903 and was 0.921, and 0.809, respectively, for the two dimensions of the CSQ.

### Statistical Analysis

Quantitative data are presented as mean ± standard deviation, and categorical data are presented as number (percentage, %). Group differences were examined by chi-square test for categorical data and analysis of variance (ANOVA) for continuous data with normal distribution. Multiple comparison of means was performed using the SNK-q test. To reduce bias for group comparisons, propensity score matching (PSM) was applied. Logistic regression was used to investigate factors associated with sleep quality. All statistical analyses were performed using SPSS. The significance level was 0.05 for all statistical tests.

## Result

### Characteristics of the Participants

Xiangya Hospital has a total of 5,562 employees. After eliminating the questionnaires that were completed in <100 s and those for which 70% of the answers were identical, a total of 4,245 respondents from Xiangya Hospital completed the survey and submitted the questionnaires (response rate: 76.3%). As shown in Table 1, there were 868 doctors (20.4%), 2,131 nurses (50.2%), 128 pharmacists (3.0%), 368 technicians (8.7%), 395 administrators (9.3%), 99 scientific researchers (2.3%), 155 logisticians (3.7%), and 101 other staffs (2.4%). With respect to working status, 2,749 (64.8%) were working in the non-epidemic area of Xiangya Hospital, 263 (6.2%) were working in the epidemic area of the hospital, 86 (2%) were assigned to the ICU of Union Hospital in Wuhan, 347 (8.2%) people were working at home, 708 (16.7%) people were resting at home, and 92 (2.2%) were quarantined.

### Description of Sleep Quality

As shown in Table 2, the prevalence of sleep disturbance among medical staffs was 37.1%. The sleep scores of the staffs assigned to the ICU of Union Hospital in Wuhan were higher than the norm (*P* = 0.003), whereas those of the staffs working at home, resting at home, and working in the non-epidemic area of Xiangya Hospital were lower than the norm (*P* < 0.001). Sleep disturbance occurred more frequently in the staffs assigned to the ICU of Union Hospital in Wuhan (58.1%), followed by quarantined staffs (43.5%) and the staffs working in the epidemic area of the hospital (41.8%). According to the number and proportion of the SRSS item score >3 points (Table 3), psychophysiological response after insomnia was the most prevalent (21.5%), followed by insufficient sleep time (18.2%), poor sleep quality (14.4%), and disrupted sleep (14.3%).

**Table 2:** The prevalence rates of sleep disturbance and SRSS scores compared with the norm.

**Working status**	**Total number**	**Sleep disturbance, *n* (%)**	**SRSS score**	**Normalized score**
Non-epidemic area of Xiangya	2,749	1,042 (37.9%)	21.1 ± 6.48^**^	22.14 ± 5.48
Epidemic area of Xiangya	263	110 (41.8%)	21.86 ± 6.81	22.14 ± 5.49
ICU in Wuhan	86	50 (58.1%)	24.28 ± 6.67^*^	22.14 ± 5.50
Working at home	347	86 (24.8%)	19.14 ± 5.65^**^	22.14 ± 5.51
Resting at home	708	249 (35.2%)	20.76 ± 6.53^**^	22.14 ± 5.52
Quarantine	92	40 (43.5%)	23.67 ± 7.39	22.14 ± 5.53
Total	4,245	1,577 (37.1%)	21 ± 6.49^**^	22.14 ± 5.56

*SRSS, Self-Rating Scale of Sleep; ICU, intensive care unit*.

* *P < 0.05*,

** *P < 0.001*.

**Table 3:** The number and percentage (%) of SRSS item score > 3.

**SRSS item**	**Score>3, *n***	**Proportion, %**
Insufficient sleep time	773	18.2
Poor sleep quality	613	14.4
Daytime sleepiness	370	8.7
Sleep hours	120	2.8
Difficulties in getting asleep	429	10.1
Disrupted sleep	605	14.3
Early awakening	430	10.1
Dreaminess or nightmares/night terrors	262	6.2
Medication	75	1.8
Psychophysiologic response after insomnia	912	21.5

*SRSS, Self-Rating Scale of Sleep*.

### Comparison by Working Status and Staff Type

Subgroup analysis was conducted for the staffs who were assigned to the ICU of Union Hospital in Wuhan (exposure group) and the staffs who were working in the epidemic area of Xiangya Hospital (control group). Six demographic variables, including gender, age, marital status, educational level, years of working, and staff type, were matched by PSM for the purpose of comparison. Before matching, educational level, years of working, and staff type were different between the two groups, whereas after matching, no differences were identified (Supplementary Table 1). There was no statistically significant difference in the mean SRSS scores between the exposure (24.36 ± 6.75) and control groups (23.23 ± 6.40) (*P* = 0.286). The prevalence of sleep disturbance of the two groups was 57.7 and 53.8%, respectively, after the match, but the difference was not statistically significant (*P* = 0.629). Due to the lack of significant difference, we further combined the two aforementioned groups as the battlefront staffs (exposure group) and made a comparison with the staffs who were working in the non-epidemic area (control group). Demographic variables before and after PSM are shown in Supplementary Table 2. After matching, the mean SRSS scores were 22.53 ± 6.75 for the exposure group and 21.51 ± 6.61 for the control group (*P* = 0.049). The prevalence of sleep disturbance in the two groups was 46.3 and 40.9%, respectively (*P* = 0.161). SNK-q was used to compare the SRSS scores between multiple staff types. The results show that the mean SRSS score was the highest among nurses, followed by doctors and administrators (Table 4). In contrast, technicians and logisticians reported the lowest mean scores.

**Table 4:** The SRSS scores of different staff types and multiple comparison.

	**Nurse *N* = 2,131**	**Doctor *N* = 868**	**Pharmacist *N* = 128**	**Technician *N* = 368**	**Administrator *N* = 395**	**Researcher *N* = 99**	**Logistician *N* = 155**
Mean ± SD	22.2 ± 6.72	20.30 ± 6.22	19.85 ± 6.02	18.70 ± 5.59	20.34 ± 6.06	19.11 ± 5.43	18.70 ± 5.89
**Mean difference**							
Nurse		−1.902^*^	−2.348^*^	−3.496^*^	−1.861^*^	−3.089^*^	−3.497^*^
Doctor	1.902^*^		−0.447	−1.595^*^	0.041	−1.187	−1.595^*^
Pharmacist	2.348^*^	0.447		−1.148	0.488	−0.74	−1.148
Technician	3.496^*^	1.595^*^	1.148		1.635^*^	0.407	−0.001
Administrator	1.861^*^	−0.041	−0.488	−1.635^*^		−1.228	−1.636^*^
Researcher	3.089^*^	1.187	0.74	−0.407	1.228		−0.408
Logistician	3.497^*^	1.595^*^	1.148	0.001	1.636^*^	0.408	

*SRSS, Self–Rating Scale of Sleep*.

* *P < 0.05*.

### Associations of Stress, Anxiety, Depression, Social Support, and Coping Style With Sleep Quality

According to Table 5, the mean SRSS scores and prevalence of sleep disturbance were significantly higher in the group with symptoms of stress, anxiety, or depression (*P* < 0.001), but significantly lower in general than in the group with better social support and positive coping (*P* < 0.001).

**Table 5:** Comparison of SRSS scores and prevalence rates of sleep disturbance among groups with positive or negative stress, anxiety, depression, social support, or positive/negative coping.

**Group**	**Stress**	**Anxiety**	**Depression**	**Social support**	**Positive coping**	**Negative coping**
**Mean SRSS score**						
Positive group	26.50 ± 6.68	25.96 ± 6.24	26.14 ± 6.64	19.28 ± 6.07	19.91 ± 6.19	21.44 ± 6.80
Negative group	19.85 ± 5.83	19.25 ± 5.61	19.77 ± 5.81	21.99 ± 6.52	23.14 ± 6.52	20.84 ± 6.37
*t*	27.371^**^	33.174^**^	27.482^**^	13.352^**^	15.762^**^	2.667^**^
**Sleep disturbance (%)**						
Positive group	71.9	68.5	69.5	26.7	30.5	38.7
Negative group	29.9	26.1	29.4	43.1	50.1	36.6
χ^2^	458.134^**^	629.301^**^	457.661^**^	112.852^**^	156.148^**^	1.664

*SRSS, Self-Rating Scale of Sleep*.

* *P < 0.05*,

** *P < 0.001*.

### Multivariable Analysis for Sleep Disturbance Among Battlefront Staffs

We constructed a logistic model with a step-back method for all first-line staffs to investigate the factors influencing sleep disturbance. Taking sleep quality as dependent variable and gender, age, educational level, years of working, professional title, disease history, living situation, exposure to confirmed or suspected COVID-19 cases, average monthly income, personal economic impact form the outbreak, recognition of the effectiveness of protection, concerns about insufficient supplies of protective materials, stress level, anxiety level, depression level, degree of positive coping, negative coping, and the level of social support as independent variables, we identified several significant factors affecting sleep disturbance (Table 6), including professional title (OR = 0.65; *P* = 0.043), anxiety (OR = 1.99; *P* < 0.001), depression (OR = 1.69; *P* = 0.035), and lesser degree of positive coping (OR = 0.44; *P* = 0.003).

**Table 6:** Logistic regression on factors for sleep disturbance among battlefront staffs.

**Factors**	**OR**	**95% CI**	***P***
**First-line staffs**			
Professional title	0.65	0.42, 0.99	0.043
Anxiety	1.99	1.50, 2.64	<0.001
Depression	1.69	1.04, 2.74	0.035
Less positive coping	0.44	0.26, 0.75	0.003
**Nurses**			
Anxiety	2.10	1.62, 2.71	<0.001
Less positive coping	0.36	0.20, 0.64	<0.001
**Doctors**			
Recognition of the effectiveness of protection	0.09	0.01, 0.67	0.018
Concerns about insufficient supplies of protective materials	3.12	1.06, 9.16	0.039
Anxiety	6.35	1.89, 21.32	0.003

*OR, odds ratio; CI, confidence interval*.

We also use a multivariable logistic regression for the first-line nurses; anxiety (OR = 2.10; *P* < 0.001) and lesser degree of positive coping (OR = 0.36; *P* < 0.001) were retained in the final model (Table 6).

For the first-line doctors, the logistic model retained recognition of the effectiveness of protection (OR = 0.09; *P* = 0.018), concerns about insufficient supplies of protective materials (OR = 3.12; *P* = 0.039), and anxiety (OR = 6.35; *P* = 0.003) as the significant factors (Table 6).

Subgroup analysis was not performed for other groups owing to limited sample size.

## Discussion

The COVID-19 outbreak in China has been going on for nearly 2 months, and the situation beyond China is worsening. Medical practitioners around the world are facing a lasting epidemic event, and ensuring sleep quality is one of the prerequisites for medical workers' physical and mental health. To understand the sleep quality and its influencing factors in medical workers during the epidemic, we immediately implemented an online survey and found that 38.7% of the 4,245 medical workers in Xiangya Hospital demonstrated sleep disturbance and the sleep quality of frontline staffs was the worst. Among the staff types, the sleep quality of nurses was the worst, followed by doctors and administrators. Professional title, anxiety, depression, and coping style were identified to be associated with sleep disturbance. Among frontline nurses, anxiety and coping style were the associated factors identified. Among frontline doctors, recognition of the effectiveness of protection, concerns about insufficient supplies of protective materials, and anxiety were the associated factors identified. The overall prevalence of sleep disturbance among medical staffs in this study was 38.7%, which was consistent with the prevalence of 34.3% found among 5,496 medical staffs in 32 provinces in China (8). The staffs assigned to the ICU of Union Hospital in Wuhan (58.1%) had the highest prevalence of sleep disturbance, followed by the quarantined staffs (43.5%) and the staffs working in the epidemic area of the hospital (41.8%). The sleep quality of the frontline group, which included the staffs assigned to the ICU of Union Hospital in Wuhan and the staffs working in the epidemic area of the hospital, was worse than that of the staffs working in the non-epidemic area of the hospital; this was consistent with findings of a previous study (8). The prevalence rate of sleep disturbance among quarantined staffs was lower than that of the staffs working on the front line, which may have been because the quarantined staffs had taken protective measures before contacting confirmed or suspected cases, none of them manifested clinical symptoms, and most were quarantined at home (except for two staffs in the isolation ward). As reported previously, the frontline staffs at a high risk of infection in the severe acute respiratory syndrome (SARS) epidemic not only had chronic stress but also suffered from depression and anxiety to a greater extent (20). Similarly, through the investigation of 512 medical workers fighting against COVID-19 in China, the medical workers who had close and direct contact with infected patients exhibited higher anxiety scores than those who had not (21). Therefore, we should attach great importance to the sleep quality of frontline medical workers, and interventional measures should be taken for them. A review of SARS research recommended that employers should make every endeavor to provide a supportive environment in the workplace and promise to ensure overall support for workers who carry the greatest burden of risk, such as those who have the most contact with patients and the most prolonged exposure to virus (22). Psychological disturbances among frontline staffs during the epidemic have also been linked to workloads (23), so the working hours of frontline staffs should be reasonably arranged. Occupational roles often have an impact on psychological effects. Our study found the worst sleep quality among staff types to be for nurses, whereas the sleep quality for technicians and logisticians was better. Anxiety and coping style were also identified as significant factors in this study. Nurses not only provide treatment, nursing, and health education but also participate in patients' rehabilitation and provide support for patients and their families. During the COVID-19 epidemic, nurses are still the main medical practitioners in the treatment of confirmed or suspected cases. During the epidemic, the public and patients were prone to psychological pressure (24, 25), and the nursing work of nurse–patient communication and spiritual care became more difficult than before. A study of nurses in South Korea showed that poor sleep quality might lead to lower nurse productivity (26). As this study shows, the higher the professional title, the better the sleep quality. Therefore, nurse leaders and managers should consider taking measures to improve nurses' sleep quality and work efficiency. Considering that fear may be more detrimental than SARS coronavirus 2 (SARS-CoV-2) in containing the COVID-19 epidemic, what counts is to avoid public panic (27). According to national policies, the administrative departments of hospitals should implement public management measures in place, such as public information dissemination, infection prevention and control training, and psychological assistance, etc., and the workload and pressure of administrators should also increase. Compared with frontline nurses, hospital managers should strengthen epidemic prevention and control knowledge training for doctors and ensure sufficient supplies of protective materials. Frontline staffs with anxiety and depression were more likely to develop sleep disturbance, which is consistent with many studies during the outbreak (7, 8). Studies of other populations have also shown that poor sleep quality was directly associated with anxiety and depressive symptoms (28, 29). Systematic epidemic prevention programs, such as in-service training, human resources allocation, gathering sufficient protective equipment, and establishing a mental health team, could improve anxiety, depression levels, and sleep quality among battlefront staffs (30). Progressive muscle relaxation training could alleviate anxiety and improve sleep quality, and this training can be performed remotely and multiple times after one training session (31). During the outbreak, we advocate that medical institutions adopt systematic prevention programs and psychological interventions to alleviate anxiety and depression and improve sleep quality. Many studies have shown that social support is a protective factor for sleep quality. Social support was significant for self-reported sleep in a study of American adults (32). In light of the study conducted by Xiao using the structural equation analysis, the levels of anxiety, stress, and self-efficacy of Chinese medical workers who had been treating patients infected by COVID-19 between January and February 2020 depended on their sleep quality and the social support they had received (33). Liu holds the opinion that the mental health, general health, physical pain, and vitality of medical workers who fought against SARS-CoV-2 pneumonia in China showed a close connection with support from family members, friends, and society as a whole (34). Therefore, medical institutions should protect their medical staffs with protective materials and provide psychological support and train them to seek help and support from their families, colleagues, and friends. This study shows that battlefront staffs with better coping skills suffered a lower risk of sleep disturbance. In the H1N1 epidemic in Japan, staff members with close ties to psychiatric services in hospitals felt less significant psychological impact, whereas those provided with less information about the pandemic felt an overwhelming sense of insecurity regarding exposure without protection (35). Therefore, support for medical staffs during the epidemic should also include knowledge dissemination and infection control training, which might also be beneficial to their coping strategies. In this study, we found that the psychophysiological response following insomnia was the most prevalent symptom of sleep disruption, and that insufficient sleep time, poor sleep quality, and disrupted sleep accounted for the largest proportions of high score sleep factors among medical staffs. Since the rest time of medical staffs was limited during the epidemic period, we can provide some feasible psychological treatment methods to improve sleep quality. By tapping into the power of music, Lestarini discovered that soft and soothing music could be used as an intervention therapy to alleviate symptoms of depression and improve sleep quality for senior citizens (36). According to a meta-analysis, through online communication, cognitive behavioral therapy was able to relieve insomnia, improve sleep efficiency and subjective sleep quality, and reduce the number of wake-ups after sleep onset and nighttime awakenings following treatment, thereby reducing latency to sleep and increase total sleep time (37). Ong et al. conceded that mindfulness intervention can effectively reduce cognitive emotional arousal associated with insomnia and increase positive emotions at a level similar to that of standard behavioral therapy for insomnia (38). Therefore, medical institutions can provide relaxation therapy audio, mindfulness audio, network cognitive behavioral therapy, etc., to help medical staffs improve sleep.

## Limitation

There are several limitations in this study. First, the generalizability might be limited in a single-centered study. Second, the sample size of the staffs assigned to Wuhan was relatively small. Third, we did not conduct face-to-face interviews, but rather an online questionnaire survey was used instead for safety and feasibility. Item response might be biased by extreme response style.

## Conclusion

Sleep disturbance is prevalent among medical staffs during the COVID-19 outbreak, especially in high-risk staffs who directly contact patients on the battlefront, as well as nurses, doctors, and administrative groups. Anxiety and depression are associated with sleep problems, whereas coping ability is a protective factor for sleep. Medical institutions should use practical psychotherapeutic methods to improve employees' psychological and sleep status.

## Data Availability Statement

The raw data supporting the conclusions of this article will be made available by the authors, without undue reservation.

## Ethics Statement

The studies involving human participants were reviewed and approved by The Ethics Committee of Xiangya Hospital, Central South University. The patients/participants provided their written informed consent to participate in this study.

## Author Contributions

JL, QZ, and FJ were responsible for the study design. SL and FG were responsible for collecting data. MS, FJ, and YX were involved in the statistical analysis. FJ was involved in the manuscript preparation and drafting the paper. HD and ZG were involved in editing and revising the manuscript. MS, SX, JL, and QZ were responsible for the critical revision of the manuscript. All authors have contributed to and have approved the final manuscript.

## Conflict of Interest

The authors declare that the research was conducted in the absence of any commercial or financial relationships that could be construed as a potential conflict of interest.

## Abbreviations

COVID: coronavirus disease
SRSS: Self-Rating Scale of Sleep
DASS: Depression, Anxiety and Stress Scale
SSRS: Social Support Rating Scale
CSQ: Simplified Coping Style Questionnaire
ICU: intensive care unit.
