# Supplementary material for: Sleep Quality in Medical Staffs During the Outbreak of Coronavirus Disease 2019 in China: A Web-Based Cross-Sectional Study
Source: Front Psychiatry. 2021 Jun 9;12:630330. doi: 10.3389/fpsyt.2021.630330 (PMC8221287; doi:10.3389/fpsyt.2021.630330)
Supplement: Supplementary file 1 [file Table_1.docx]

**Supplementary Table 1. Comparison of general demography of the staffs assigned to the ICU of Union Hospital in Wuhan and the staffs working in the epidemic area of Xiangya hospital before and after propensity score matching**

| Variable | Before matching | | |  | After matching | | |
| --- | --- | --- | --- | --- | --- | --- | --- |
|  | ICU in Wuhan  (n=86) | Epidemic area of  Xiangya Hospital (n=263) | *P* |  | ICU in Wuhan  (n=78) | Epidemic area of  Xiangya Hospital (n=78) | *P* |
| Gender |  |  | 0.251 |  |  |  | 0.616 |
| Male | 14 (16.3%) | 58 (22.1%) |  |  | 10(12.8%) | 8(10.3%) |  |
| Female | 72(83.7%) | 205(77.9%) |  |  | 68(87.2%) | 70(89.7%) |  |
| Age, n% |  |  | 0.102 |  |  |  |  |
| ＜31 | 49 (57.0%) | 128 (48.7%) |  |  | 46(59.0%) | 48(61.5%) | 0.822 |
| 31-40 | 35 (40.7%) | 107(40.7%) |  |  | 30(38.5%) | 29(37.2%) |  |
| 41-50 | 2 (2.3%) | 24(9.1%) |  |  | 2(2.6%) | 1(1.3%) |  |
| ＞50 | 0 (0.0%) | 4(1.5%) |  |  |  |  |  |
| Marital status |  |  | 0.982 |  |  |  | 1.000 |
| Unmarried | 34 (39.5%) | 101 (38.4%) |  |  | 30(38.5%) | 30(38.5%) |  |
| Married | 51 (59.3%) | 159 (60.5%) |  |  | 48(61.5%) | 48(61.5%) |  |
| Divorced | 1 (1.2%) | 3 (1.1%) |  |  |  |  |  |
| Education |  |  | 0.003 |  |  |  | 0.972 |
| Junior college | 7 (8.1%) | 17 (6.5%) |  |  | 7(9.0%) | 7(9.0%) |  |
| Undergraduate | 67 (77.9%) | 159 (60.5%) |  |  | 62(79.5%) | 62(79.5%) |  |
| Master | 8 (9.3%) | 30 (11.4%) |  |  | 5(6.4%) | 6(7.7%) |  |
| Doctor | 4 (4.7%) | 57 (21.7%) |  |  | 4(5.1%) | 3(3.8%) |  |
| Staff type |  |  | 0.001 |  |  |  | 1.000 |
| Nurse | 81(94.2%) | 183(69.6%) |  |  | 74(94.9%) | 74(94.9%) |  |
| Doctor | 4(4.7%) | 63(24.0%)) |  |  | 4(5.1%) | 4(5.1%) |  |
| Technician | 1(1.2%) | 8(3.0%) |  |  |  |  |  |
| Administrator | 0(0.0%) | 4(1.5%) |  |  |  |  |  |
| Researcher | 0(0.0%) | 3 (1.1%) |  |  |  |  |  |
| Logistician | 0(0.0%) | 1 (0.4%) |  |  |  |  |  |
| Other | 0(0.0%) | 1 (0.4%) |  |  |  |  |  |
| Working years |  |  | 0.004 |  |  |  | 0.998 |
| ≤5 | 24 (27.9%) | 108(41.1%) |  |  | 23(29.5%) | 24(30.8%) |  |
| 6-10 | 48(55.8%) | 88(33.5%) |  |  | 42(53.8%) | 41(52.6%) |  |
| 11-20 | 12(14.0%) | 45(17.1%) |  |  | 11(14.1%) | 11(14.1%) |  |
| 21-30 | 2(2.3%) | 20(7.6%) |  |  | 2(2.6%) | 2(2.6%) |  |
| ≥31 | 0(0.0%) | 2(0.8%) |  |  |  |  |  |

Note: ICU, intensive care unit.
