# Supplementary material for: Sleep Quality in Medical Staffs During the Outbreak of Coronavirus Disease 2019 in China: A Web-Based Cross-Sectional Study
Source: Front Psychiatry. 2021 Jun 9;12:630330. doi: 10.3389/fpsyt.2021.630330 (PMC8221287; doi:10.3389/fpsyt.2021.630330)
Supplement: Supplementary file 2 [file Table_2.docx]

**Supplementary Table2. Comparison of general demography of the staffs working on the front line and the staffs working in the non-epidemic area of Xiangya hospital before and after propensity score matching**

| Variable | | Before matched | | |  | After matched | | |
| --- | --- | --- | --- | --- | --- | --- | --- | --- |
|  |  | Front line (n=349) | Non-epidemic area (n=2749) | *P* |  | Front line (n=335) | Non-epidemic area (n=335) | *P* |
| Gender | |  |  | 0.113 |  |  |  | 0.769 |
| Male | | 72 (20.6%) | 673 (24.5%) |  |  | 63 (18.8%) | 66(19.7%) |  |
| Female | | 277 (79.4%) | 2076 (75.5%) |  |  | 272 (81.2%) | 269(80.3%) |  |
| Age | |  |  | <0.001 |  |  |  | 0.974 |
| ＜31 | | 177 (50.7%) | 849 (30.9%) |  |  | 173(51.6%) | 173(51.6%) |  |
| 31-40 | | 142 (40.7%) | 1201 (43.7%) |  |  | 133(39.7%) | 130(38.8%) |  |
| 41-50 | | 26 (7.4%) | 491 (17.9%) |  |  | 25(7.5%) | 27(8.1%) |  |
| >50 | | 4(1.1%) | 207 (7.5%) |  |  | 4(1.2%) | 5(1.5%) |  |
| Marriage Status | |  |  | <0.001 |  |  |  | 0.402 |
| Unmarried | | 135 (38.7%) | 520 (18.9%) |  |  | 127(37.9%) | 127(37.9%) |  |
| Married | | 210 (60.2%) | 2154 (78.4%) |  |  | 204(60.9%) | 207 (61.8%) |  |
| Divorced | | 4 (1.1%) | 75 (2.7%) |  |  | 4(1.2%) | 1 (0.3%) |  |
| Educational level | |  |  | <0.001 |  |  |  | 0.974 |
| High school and below | | 0 (0.0%) | 39 (1.4%) |  |  |  |  |  |
| Junior college | | 24 (6.9%) | 246 (8.9%) |  |  | 22(6.6%) | 22(6.6%) |  |
| Undergraduate | | 226 (64.8%) | 1370 (49.8%) |  |  | 221(66.0%) | 222(66.3%) |  |
| Master | | 38 (10.9%) | 532 (19.4%) |  |  | 33(9.9%) | 34(10.10%) |  |
| Doctor | | 61 (17.5%) | 562 (20.4%) |  |  | 59(17.6%) | 57(17.0%) |  |
| Staff type | |  |  | <0.001 |  |  |  | 0.939 |
| Nurse | | 264 (75.6%) | 1306 (47.5%) |  |  | 225(76.1%) | 225(76.1%) |  |
| Doctor | | 67 (19.2%) | 599 (21.8%) |  |  | 65(19.4%) | 64(19.1%) |  |
| Pharmacists | | 0 (0.0%) | 94 (3.4%) |  |  | 0(0.0%) | 1(0.3%) |  |
| Technician | | 9 (2.6%) | 209 (7.6%) |  |  | 6(1.8%) | 4 (1.2%) |  |
| Administrator | | 4 (1.1%) | 302 (11.0%) |  |  | 4(1.2%) | 6 (1.8%) |  |
| Researcher | | 3 (0.9%) | 35 (1.3%) |  |  | 3 (0.9%) | 2 (0.6%) |  |
| Logistician | | 1 (0.3%) | 134 (4.9%) |  |  | 1 (0.3%) | 2 (0.6%) |  |
| Other | | 1 (0.3%) | 70 (2.5%) |  |  | 1 (0.3%) | 1 (0.3%) |  |
| Working years | |  |  | <0.001 |  |  |  | 0.999 |
| ≤5 | | 132 (37.8%) | 669 (24.3%) |  |  | 127(37.9%) | 130(48.8%) |  |
| 6-10 | | 136 (39.0%) | 964(35.1%) |  |  | 13 (39.1%) | 128(38.2%) |  |
| 11-20 | 57 (16.3%) | | 568(20.7%) |  |  | 54 (16.1%) | 54 (16.1%) |  |
| 21-30 | 22 (6.3%) | | 402(14.6%) |  |  | 21 (6.3%) | 21 (6.3%) |  |
| ≥31 | 2 (0.6%) | | 146(5.3%) |  |  | 2(0.6%) | 2(0.6%) |  |
